# Contextualized acceleration and deceleration profiles of elite soccer players during English Premier League match-play. The effect of possession, positional demands and opponent ranking

**DOI:** 10.5114/biolsport.2025.148540

**Published:** 2025-04-14

**Authors:** Ryland Morgans, Mauro Mandorino, Ben Ryan, Piotr Zmijewski, Alexandre Moreira, Rafael Oliveira

**Affiliations:** 1 School of Sport and Health Sciences, Cardiff Metropolitan University, Cardiff, UK; 2 Brentford FC Football Research Centre, Brentford FC, London, UK; 3 Performance and Analytics Department, Parma Calcio 1913, 43121 Parma, Italy; 4 Department of Movement, Human and Health Sciences, University of Rome “Foro Italico”, 00135 Rome, Italy; 5 Jozef Pilsudski University of Physical Education in Warsaw, 00-809 Warsaw, Poland; 6 Research and Development Center Legia Lab, Legia Warszawa, Poland; 7 Department of Sport, School of Physical Education and Sport, University of São Paulo, São Paulo, Brazil; 8 Research Centre in Sports Sciences, Health and Human Development, (CIDESD), Santarém Polytechnic University, 2040-413 Rio Maior, Portugal; 9 Santarém Polytechnic University, School of Sport, 2040-413 Rio Maior, Portugal

**Keywords:** Contextual variables, Optical tracking, Match performance, Football, Elite soccer

## Abstract

This study aimed to compare accelerations (ACC) and decelerations (DEC) when in- (_IP_) and outof-possession (_OP_) during official English Premier League (EPL) match-play over three consecutive seasons considering playing position, match location and opponent ranking. Match data from 31 male professional soccer players was obtained via an optical tracking system. Playing position significantly influenced ACC_IP_, DEC_IP_, and DEC_OP_. Ranking difference and match location were significant predictors for DEC_IP_, ACC_OP_, and DEC_OP_. An increase in ranking difference was associated with higher DEC_IP_. Conversely, in the out-of-possession phase (ACC_OP_ and DEC_OP_), a decrease in these parameters with increasing ranking difference was observed. Regarding match location, positive β coefficients suggested that DEC_IP_, ACC_OP_, and DEC_OP_ values were significantly higher during away matches compared to home matches. A significant interaction (playing position × ranking difference) was reported for DEC_OP_ (β = 0.035, p = 0.013). Interestingly, DEC_OP_ actions decreased with increasing ranking difference across all playing positions except for wingers. In conclusion, these findings highlight that distinct contextual factors influence ACC and DEC performance during in- and out-of-possession phases of EPL match-play.

## INTRODUCTION

High-intensity actions such as accelerating and decelerating have recently shown to significantly influence decisive moments of the match [1–3]. Consequently, high-intensity movements in match-play have gained more attention [2, 4].

These high-intensity actions induce not only physiological but also mechanical demands, accounting for ~10% of total workload of elite soccer players during match-play, irrespective of playing position [5–7]. Additionally, the number of accelerations (ACC) during match-play is up to ~8 times higher than sprint actions (~90–120 vs ~15–30, respectively) [6, 7], while deceleration (DEC) actions occur as frequently as ACC activities, leading to an even greater mechanical load [5]. Thus, it appears crucial for practitioners to profile such explosive actions throughout a match and season so that players can be prepared to cope with the physiological and mechanical demands of match-play [2, 3, 8, 9]. However, there is scant literature examining ACC and DEC in elite soccer [10], thus underscoring the pertinence of the current study as it analyzed possession, positional differences and opponent ranking, which are considered key contextual factors [11].

Performance match demands in the English Premier League (EPL) are complex with numerous influences on player outputs [2, 3, 8, 9, 11–14]. Contextual variables including possession, positional differences, opposition ranking, team formation, and match location have recently been observed as key contributors to performance [2, 9, 12, 13]. Players need to execute explosive actions, such as ACC and DEC while under pressure during in- and out-of-possession [13, 15, 16]. Therefore, the context in which these high-intensity match actions occur is vital to fully understand how to optimally prepare players for positional demands and the game [2, 3, 8, 9, 11–14]. For example, full-backs (FB) performed 22% more ACC than wide midfielders (WM), while all players performed 6% more ACC actions during home matches compared to away fixtures [2]. However, DEC efforts seem to only be affected by position, with FB and centre midfielders (CM) executing 26% and 32% greater DEC efforts than centre backs (CB), respectively. Furthermore, when playing against top or middle teams at home, CB, CM, and centre forwards (CF) tended to perform more high-intensity actions than when playing away [2]. In addition, some studies showed that central midfielders performed more moderate-intensity ACC distance when compared to attackers and defenders, among elite academy EPL [17] and Spanish First Division [18] soccer players. It has also been verified that wide attackers and wide defenders have shown the highest values of high-intensity ACC due to the repeated attacking and defensive functions of these positions in the EPL [17].

Other factors, such as opponent ranking, formation and in- and out-of-possession tactical strategies [2, 9, 12, 13, 15], also contribute to the physical demands placed on players during elite level matchplay [14, 19]. In contrast, when considering match location and result, a recent study did not report any effect on physical performance during official competition [20]. Furthermore, other tactical-contextual factors (i.e., phases of play, attacking/defensive organization and transitions) have been investigated to verify the physical effect during matches [21, 22]. Although, these studies did not examine the influence of tactical-contextual factors on explosive actions, such as ACC and DEC, thus limiting the generalization of the results to other teams and leagues. Therefore, further research is warranted.

Recently, increased physical demands against high-ranked opponents have been found [9, 12, 14, 16, 19, 23]. These results emphasize the requirement of players and teams to tactically and physically adjust according to differing problems proposed by quality opponents [9, 12, 14, 16, 19, 23]. Furthermore, understanding the fluctuations in physical outputs across different levels of opposition quality (low-, middle- and high-ranked teams) can provide invaluable information to support effective training and recovery strategies [9, 12, 14, 16, 19, 23].

Currently there is limited evidence examining contextualized ACC and DEC actions during elite-level match-play in relation to possession, players’ position and opponent ranking, thus the present study would seem to present real-world practical significance. The aims of this study were, firstly, to compare ACC and DEC when in- and outof-possession during official EPL match-play over three consecutive seasons; and secondly, to examine any differences considering playing position, match location and opponent ranking. Based on existing literature [2, 24], the study hypothesis was that positional differences of ACC and DEC would be evident, with a higher number of ACC and DEC observed when out-of-possession during EPL matchplay and that match location and opponent ranking would also influence ACC and DEC actions.

## MATERIALS AND METHODS

### Study Design

A retrospective study was conducted analyzing EPL match data from the 2021–2022 to 2023–2024 seasons for a cohort of 31 male professional soccer players. Data were collected via an optical tracking system from 20 EPL stadiums.

This research utilized a three-year longitudinal study design. A nonprobabilistic sampling protocol was employed to recruit the participants. The emphasis of the study was on monitoring player ACC and DEC while in- and out-of-possession during competitive EPL matchplay. During the observational seasons 2021–2022 to 2023–2024, consistent player monitoring approaches were implemented without any interference from the researchers [3].

### Participants

Thirty-one professional first-team squad outfield soccer players from an EPL club were involved in the study (age 24.6 ± 5.4 years, weight 76.6 ± 6.9 kg, height 1.79 ± 0.09 m). The EPL team adopted a 4-3-3 or 3-5-2 formation and implemented a hybrid model of possession that included a combination of build-up and direct-play strategies [3]. Furthermore, when out-of-possession a mixture of high-press and mid-block (a narrow and compact team shape defending the middle third of the pitch) strategies were employed.

The research inclusion criteria have previously been applied [2, 3, 8, 9, 11–13] and were: (i) named in the first-team squad at the start of all study seasons, (ii) played in at least 80% of matches, (iii) only completed official team training during the study period, and (iv) completed at least 90-minutes of match-play. Additionally, the exclusion criteria for the study have also been previously employed [2, 3, 8, 9, 11–13] and included: (i) long-term injury (three months or longer), (ii) joining the team late in any of the study seasons, (iii) lack of full, complete match data, and (iv) goalkeepers, due to the variations in physical demands [21].

Players were classified as: centre-backs (CB; n = 10), full-backs (FB; n = 11), centre midfielders (CM; n = 8), wingers (W; n = 5), and centre forwards (CF; n = 7). If a player fulfilled multiple playing positions during match-play, the player was categorized accordingly to each position. Considering the high variability of ACC and DEC profiles, only players who completed 90-minutes were considered. This did not affect the sample size or statistical power. The small sample size is supported by previous studies in soccer [1–6, 20]. Even so, the power of the sample size was calculated through G-Power [25]. Post-hoc analysis was conducted considering the study aims. For comparison analysis, an F-test, with a total of 31 participants with a *p* = 0.05 and effect size of 0.2 was performed. The actual power achieved was 95.4%.

All data collected resulted from normal analytical procedures regarding player monitoring over the competitive season [2, 3, 8, 9, 11–13], nevertheless, written informed consent was obtained from all participants. The study was conducted according to the requirements of the Declaration of Helsinki and was approved by the local Ethics Committee of Cardiff Metropolitan University and the club from which the participants volunteered [26]. To ensure confidentiality, all data were anonymized prior to analysis.

### Data Collection

Data were collected during all (n = 114) regular-season EPL competitive matches played by the examined team across the three study seasons (2021–2022 to 2023–2024).

League match data across the study seasons were recorded and analyzed via the optical tracking system Second Spectrum to report physical performance data. Second Spectrum has been validated by the FIFA program to meet industry standards [27]. Data were collected via semi-automated HD cameras positioned around the stadium with a sampling frequency of 25 Hz.

Variables analyzed were selected based on previous publications [16, 28] and were analyzed as absolute (total number) and relative data (divided by actual playing time for each player). Thus, the total number of ACC and DEC and the number of ACC (> +3 m/s^-2^ with a minimum duration of 0.5 s) and DEC (< -3 m/s^-2^ with a minimum duration of 0.5 s) per minute [29] were examined. The following tactical/physical variables were also quantified in this study: the number of ACC when in-possession (ACC_IP_); the number of DEC when in-possession (DEC_IP_); the number of ACC when opposition inpossession (ACC_OP_); and the number of DEC when opposition in-possession (DEC_OP_) [16].

Each match was classified based on the ranking difference between the study team and opponent at that specific point in the season. This ranking difference was treated as a continuous variable, reflecting the number of positions separating the two teams in the league standings [16]. A positive value indicated that, at that moment in the season, the team held a higher (better) rank than its opponent, whereas a negative value signified that the team was ranked lower (worse) than its opponent [16].

The Second Spectrum match data were processed directly using the Python programming language (Python 2.7) through the Spyder scientific development environment (https://www.spyderide.org/). Processing data directly via programs such as Python 2.7 allows more detailed analysis of the differing phases. Publishing the exact algorithms used to determine the examined metrics was not possible due to the intellectual property rights of the technological commercial entities [30]. Thus, the specific conversion and filtering algorithms utilized in these systems were not available.

### Statistical Analysis

A two-level multilevel regression analysis was conducted to assess the influence of playing position, ranking difference, and match location (home/away) on ACC and DEC during possession (ACC_IP_, DEC_IP_) and out-of-possession phases (ACC_OP_, DEC_OP_). Level 1 comprised of player position, while Level 2 included contextual factors such as ranking difference and match location.

Ranking difference was treated as a continuous variable, whereas playing position (CB = 0, FB = 1, CM = 2, W = 3, CF = 4) and match location (home = 0, away = 1) were treated as ordinal variables. Independent variables were modelled as fixed effects, while players were treated as random effects to account for intra-player correlations due to repeated measurements.

The model-building process followed a stepwise approach for each dependent variable: – Model 1: Included the Level 1 predictor (playing position).

– Model 2: Added Level 2 predictors (ranking difference and match location).
– Model 3: Included the interaction between playing position and ranking difference.
– Model 4: Included the interaction between playing position and match location.
– Model 5: Included the interaction between ranking difference and match location.

Assumptions of normality and homogeneity of variance were tested prior to conducting the multilevel regression analysis. Normality was evaluated using QQ plots, while Bartlett’s test was employed to assess homogeneity of variance, ensuring the validity of the model assumptions. Model fit was assessed using the Akaike Information Criterion (AIC), with lower AIC values indicating better fit. When significant differences were identified in the multilevel regression analysis for playing position, the least significant difference (LSD) approach to multiple comparisons was applied, as recommended by [31]. Standardized effect sizes (ES), calculated as the mean difference divided by the pooled standard deviation, were interpreted as small (0.2), moderate (0.5), or large (0.8). All statistical analyses were performed using IBM SPSS Statistics, version 28.0 (IBM Corp., Armonk, NY, USA). Statistical significance was set at *p* < 0.05.

## RESULTS

**FIG. 1:**
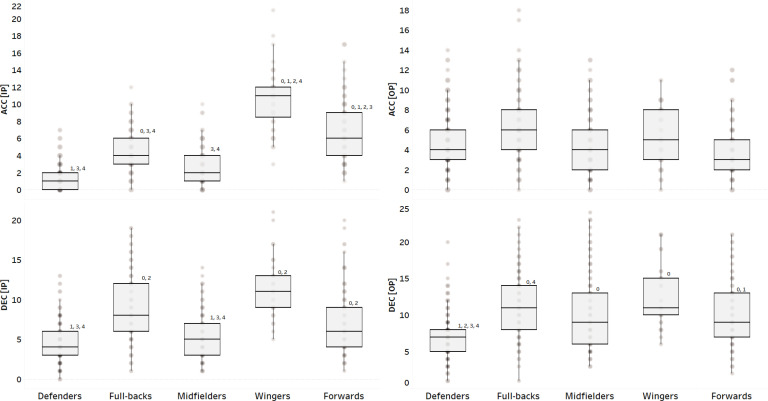
Comparison of ACC_IP_, DEC_IP_, ACC_OP_, and DEC_OP_ according to playing position. **ACC_IP_** = count of accelerations in-possession phase; **DEC_IP_** = count of decelerations in-possession phase; **ACC_OP_** = count of accelerations out-of-possession phase; **DEC_OP_** = count of decelerations out-of-possession phase; **ES** = effect size; **0** denotes significant difference vs. Defenders; **1** denotes significant difference vs. Full-backs; **2** denotes significant difference vs. Midfielders (*p* < 0.05); **3** denotes significant difference vs. Wingers (*p* < 0.05); **4** denotes significant difference vs. Forwards (*p* < 0.05). **ACC**_IP_ ES (Defenders vs. Full-backs) = 1.47 ES (Defenders vs. Wingers) = 3.29 ES (Defenders vs. Forwards) = 1.99 ES (Full-backs vs. Wingers) = 2.00 ES (Full-backs vs. Forwards) = 0.80 ES (Midfielders vs. Wingers) = 2.87 ES (Midfielders vs. Forwards) = 1.50 ES (Wingers vs. Forwards) = 1.12 **ACC**_OP_ No significant differences **DEC**_IP_ ES (Defenders vs. Full-backs) = 1.27 ES (Defenders vs. Wingers) = 2.39 ES (Defenders vs. Forwards) = 2.32 ES (Full-backs vs. Wingers) = 1.32 ES (Full-backs vs. Midfielders) = 1.04 ES (Midfielders vs. Wingers) = 1.07 ES (Midfielders vs. Forwards) = 0.59 **DEC**_OP_ ES (Defenders vs. Full-backs) = 1.13 ES (Defenders vs. Wingers) = -0.74 ES (Defenders vs. Forwards) = -0.78 ES (Full-backs vs. Wingers) = -1.73 ES (Full-backs vs. Forwards) = -0.30

**FIG. 2:**
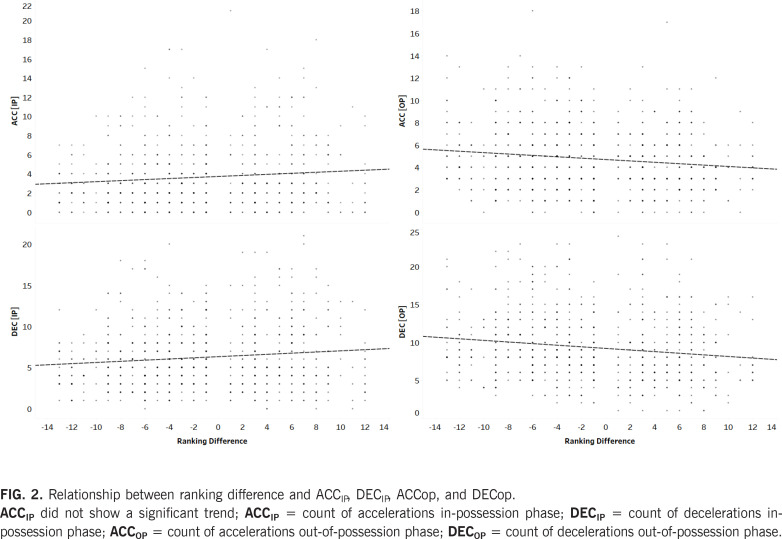
Relationship between ranking difference and ACC_IP_, DEC_IP_, ACCop, and DECop. **ACC_IP_** did not show a significant trend; **ACC_IP_** = count of accelerations in-possession phase; **DEC_IP_** = count of decelerations inpossession phase; **ACC_OP_** = count of accelerations out-of-possession phase; **DEC_OP_** = count of decelerations out-of-possession phase.

**FIG. 3:**
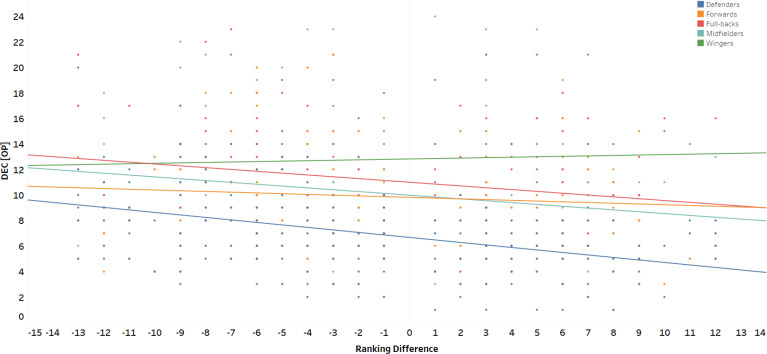
Interaction between playing position and ranking difference for the DECop.

The results of the multilevel regression analysis are summarized in Table 1, which presents the β coefficients, *p*-values, and AIC values for each model. Box plots were used to illustrate the distribution, central tendency (median), and spread (interquartile range) of ACC_IP_, DEC_IP_, ACC_OP_, and DEC_OP_ across different playing positions and match locations. Model 2 was identified as the best-fitting model for most variables, except for DEC_OP_, where Model 3 exhibited the lowest AIC value (3604.960). Playing position significantly influenced ACC_IP_, DEC_IP_, and DEC_OP_, with ES and differences shown in Figure 1. Ranking difference and match location were significant predictors for DEC_IP_, ACC_OP_, and DEC_OP_. A positive β coefficient for ranking difference indicated that an increase in ranking difference was associated with higher DEC_IP_. Conversely, during the out-of-possession phase (ACC_OP_ and DEC_OP_), a negative β coefficient was observed, indicating a decrease in these parameters with increasing ranking difference (Figure 2). Regarding match location, positive β coefficients suggested that DEC_IP_, ACC_OP_, and DEC_OP_ values were significantly higher during away matches compared to home matches. The only significant interaction in Model 3 (playing position × ranking difference) was observed for DEC_OP_ (β = 0.035, *p* = 0.013). Interaction effects and slopes for different playing positions are presented in Figure 3. Interestingly, DEC_OP_ actions decreased with increasing ranking difference across all playing positions except for W.

**TABLE 1:** Multilevel regression analysis of accelerations (ACC_IP_, ACC_OP_) and decelerations (DEC_IP_, DEC_OP_) per minute according to playing position, ranking difference, match location, and the interaction.

	ACC_IP_ / min					ACC_OP_ / min				

	Model 1	Model 2	Model 3	Model 4	Model 5	Model 1	Model 2	Model 3	Model 4	Model 5
Fixed variables										

Playing position	0.732 (0.001)	0.903 (0.001)	0.926 (0.001)	0.919 (0.001)	0.920 (0.001)	0.048 (0.814)	0.089 (0.665)	0.074 (0.722)	0.179 (0.410)	0.179 (0.408)

Ranking difference	N.E.	0.021 (0.078)	0.009 (0.595)	0.009 (0.590)	0.047 (0.030)	N.E.	-0.071 (0.001)	-0.059 (0.005)	-0.061 (0.004)	-0.069 (0.010)

Match location	N.E.	0.090 (0.560)	0.087 (0.572)	0.064 (0.767)	-0.056 (0.800)	N.E.	0.506 (0.008)	0.509 (0.007)	0.829 (0.002)	0.854 (0.002)

Playing position × ranking difference	N.E.	N.E.	0.008 (0.315)	0.008 (0.325)	0.006 (0.418)	N.E.	N.E.	-0.008 (0.432)	-0.006 (0.537)	-0.006 (0.558)

Playing position × match location	N.E.	N.E.	N.E.	0.015 (0.146)	0.032 (0.757)	N.E.	N.E.	N.E.	-0.219 (0.093)	-0.222 (0.089)

Ranking difference × match location	N.E.	N.E.	N.E.	N.E.	-0.069 (0.004)	N.E.	N.E.	N.E.	N.E.	0.014 (0.629)

Goodness of fit (AIC)	3083.846	**2907.527**	2914.228	2916.847	2914.330	3387.319	**3165.142**	3171.827	3171.243	3176.197

	**DEC_IP_ / min**					**DEC_OP_ / min**				

	**Model 1**	**Model 2**	**Model 3**	**Model 4**	**Model 5**	**Model 1**	**Model 2**	**Model 3**	**Model 4**	**Model 5**

Fixed variables										

Playing position	0.514 (0.061)	0.634 (0.022)	0.658 (0.018)	0.763 (0.008)	0.763 (0.008)	1.306 (0.001)	1.405 (0.001)	1.476 (0.001)	1.598 (0.001)	1.603 (0.001)

Ranking difference	N.E.	0.043 (0.009)	0.028 (0.236)	0.025 (0.273)	0.037 (0.207)	N.E.	-0.138 (0.001)	-0.190 (0.001)	-0.193 (0.001)	-0.238 (0.001)

Match location	N.E.	0.672 (0.001)	0.669 (0.002)	0.994 (0.001)	0.956 (0.002)	N.E.	0.542 (0.040)	0.531 (0.043)	0.905 (0.015)	1.048 (0.006)

Playing position × ranking difference	N.E.	N.E.	0.010 (0.356)	0.012 (0.285)	0.011 (0.304)	N.E.	N.E.	0.035 (0.013)	0.037 (0.009)	0.039 (0.006)

Playing position × match location	N.E.	N.E.	N.E.	-0.222 (0.123)	-0.217 (0.134)	N.E.	N.E.	N.E.	-0.256 (0.155)	-0.277 (0.124)

Ranking difference × match location	N.E.	N.E.	N.E.	N.E.	-0.021 (0.517)	N.E.	N.E.	N.E.	N.E.	0.081 (0.047)

Goodness of fit (AIC)	3547.063	**3313.880**	3320.125	3319.778	3324.338	3873.429	3605.549	**3604.960**	3605.525	3606.122

## DISCUSSION

This study aimed to compare ACC and DEC when in- and out-ofpossession during official EPL match-play over three consecutive seasons; and secondly, to examine any differences considering playing position, match location, and opponent ranking. The main finding of this study was that ranking difference and match location were significant factors influencing ACC_IP_, ACC_OP_, and DEC_IP_, while playing position and ranking difference emerged as critical variables when analyzing DEC_OP_. These insights align with the results of the statistical models, where Model 2 was identified as the best-fitting model for most variables, except for DEC_OP_, where Model 3 demonstrated the lowest AIC value. This emphasizes the importance of accounting for specific contextual and positional factors when evaluating physical performance metrics in elite soccer.

The results of this study reveal key insights into the physical performance dynamics in elite EPL soccer, particularly highlighting DEC actions between in- and out-of-possession phases. Furthermore, ACC analysis should also include ranking difference and match location variables. Nonetheless, there were significant differences when analyzing playing position except for ACC_OP_, where no differences were found. Still, when examining ACC_IP,_ ACC_OP_ and DEC_IP,_ CB presented the lowest values while W presented the highest when compared against all other positions. Such findings reinforce existing evidence that suggests player tactical roles are associated with distinct movement patterns and distances and speeds during in-possession [22], which were also confirmed in the ACC and DEC findings in the present study. Considering positional differences, defenders aim to maintain team structure and retain possession. In fact, this tactical role is very similar to that CM, thus this may partly explain the comparable values for these positions in the present study. For instance, a CM may prioritize positioning to intercept opponent forward passes or prevent quick, direct transitional play, which typically results in less ACC compared to W and forwards. Although, this positioning role is crucial for overall team shape and strategy when in- and outof-possession [32, 33]. When examining the Chinese Super League, the team that produces higher possession attacks, where three lines of players move together deep into the opponents’ half, this strategy produces greater distances to cover, specifically for W [22]. However, when the higher possession team needs to defend, W must sprint back to track an opposition player or chase/press the ball until it is regained, which can result in increases in high-speed and sprint distances from the fast counterattack [22].

Regarding DEC_OP,_ the number of DEC decreased with increasing ranking difference across all playing positions, except for W. This finding aligns with results of previous research [14] indicating that players tend to adjust physical performance based on perceived competitive pressure. For example, a mid-table team facing a top-tier opponent might conserve energy by reducing high-intensity runs, especially if prolonged periods of defending out-of-possession are anticipated [14], which is consequently associated with lower DEC actions according to the data of the present study. This may lead to less effective pressing and thus increased opportunities for opponents to exploit. Notwithstanding, previous research [34] conducted in the Persian Gulf Premier League indicated that soccer players showed a tendency of higher DEC actions (< –4 m/s^–2^) against toplevel teams. Such findings were not evident for lower intensity DEC actions, although, it is relevant to note that this research examined a small sample size without the context of ball possession [34].

Match location was particularly relevant for DEC_IP_, ACC_OP_, and DEC_OP_ where significantly higher values during away matches compared to home matches were observed. While no existing research was found addressing ball possession, a recent study reported no differences when considering match location during consecutive seasons of EPL match-play [9]. However, there was a tendency for higher ACC values at home against top six and mid-table teams when compared to bottom six teams at home for FB [9], which contrasts the present findings. Moreover, if match outcome, opponent ranking and match location were considered, there were a tendency of higher values in home matches than away matches [9], which again contrasts the present findings. Furthermore, these findings should be interpreted considering the context of the analyzed team, which as previously mentioned adopted a 4-3-3 or 3-5-2 formation and implemented a hybrid model of possession that included a combination of build-up and direct-play strategies [3], while when out-ofpossession a mixture of high-press and mid-block strategies were employed.

The observed patterns for ACC_IP_ and DEC_IP_ were similar in the present study and did not show a significant difference when considering ball possession. Although, when including other contextual factors, the results differ as previously mentioned, which emphasizes the complexity of analyzing these dynamic factors. For example, a defender may focus on tactical positioning against an elite-ranked forward to support team structure, rather than performing individual pressing movements that produce ACC and DEC actions, especially if the attacking team has a superior ranking [35]. In contrast, explosive players, such as W, tend to execute high-intensity ACC and DEC actions to stretch the defence and attack, highlighting the high physical demands of these tactical roles [22, 33].

### Practical Applications

The findings of this study offer several practical applications for coaches, analysts, and sports scientists involved in elite soccer, particularly in the EPL. Potentially key applications based on the distinction between physical performance factors when in- and out-ofpossession combined with playing position, opponent ranking and match location. Consequently, coaches can design specific training regimens that address the unique demands of each playing position during different phases of possession. For instance, a tendency of decreasing DEC_OP_ for all positions (except for CF) against increasing ranked teams, supported by previous literature, showed minimal differences considering formation and possession [9], suggesting that tactical role may be more relevant to train. Considering the identical pattern observed during match-play for DEC_IP_, ACC_OP_, and DEC_OP,_ the implementation of small-sided games as part of the training method would seem to be the best approach to develop this type of action [36, 37].

Additionally, including the influence of opponent ranking difference and match location into match preparation strategies may help inform tactical preparations such as analyzing opponent strengths. Coaches may develop strategies that account for the opponent’s ranking, potentially adjusting the team’s pressing intensity. For instance, against a top-ranked team, a mid-table team might opt for a more compact defensive formation to conserve energy and aim to counterattack more effectively rather than pressing high up the pitch and leaving space behind the defensive line, that may be exploited. Finally, contextualized player profiles can be created to consider the individual role and contextual influences based on the specific tactical role assigned. For example, measuring a midfielder’s success in transitions can help in evaluating contributions more accurately.

### Limitations and Future Research Direction

While this study highlights the contextualized ACC and DEC actions of elite soccer players with reference to possession, positional demands, opponent ranking and match location, it is essential to acknowledge certain limitations. Factors such as individual player characteristics and opposition team strategies may influence these patterns and thus must be considered the principal limitation associated with the current study. However, such characteristics and context are almost impossible to include in the practice of soccer science research. In addition, the analyzed data came from one EPL team and thus must be interpreted with caution. Future research could therefore consider individual player profiles and other contextual factors (e.g., match outcome) [9] to provide a more comprehensive understanding of the intricacies involved in player physical performance against varying levels of opposition and identify any further positional differences. Future research could also explore the impact of tactical systems as other studies have highlighted the influence on match load [14, 21, 38]. Additionally, future studies should consider how differing tactical strategies, such as high press or mid/low defensive blocks, amplify or mitigate the influence of opponent ranking difference and match location on ACC and DEC metrics. Furthermore, future research could explore the inclusion of tactical factors (e.g., field tilt, territorial domination) to further understand how match effort and high-speed demands are influenced by different playing styles. The inclusion of various teams (e.g., from different countries and leagues) with different tactical philosophies or competing in the same league but with distinct physical demands would also advance knowledge in this field.

## CONCLUSIONS

This study identified that distinct contextual factors influence ACC and DEC performance during in- and out-of-possession phases during EPL match-play. Specifically, playing position significantly influenced ACC_IP_, DEC_IP_, and DEC_OP_, while opponent ranking difference and match location were significant predictors for DEC_IP_, ACC_OP_, and DEC_OP_. When considering playing position during the out-of-possession phase, defenders exhibited the lowest number of DEC, while ACC did not differ significantly. While during the in-possession phase, defenders and midfielders displayed the lowest numbers of both ACC and DEC. Additionally, during the out-of-possession phase a decrease in ACC_OP_ and DEC_OP_ with increasing opponent ranking difference was observed. Regarding match location, DEC_IP_, ACC_OP_, and DEC_OP_ values were significantly higher during away matches compared to home matches, while DEC_OP_ activity decreased with increasing opponent ranking difference across all playing positions, except for W. In conclusion, these findings highlight that by applying these insights into practical coaching strategies, teams can potentially enhance performance and adaptability across different match situations and seasons. This comprehensive understanding of how player positions and contextual factors interact during both in- and out-of-possession phases will not only improve individual player performance but may also contribute to overall team success.
